# Rehabilitation Interventions for Unilateral Neglect after Stroke: A Systematic Review from 1997 through 2012

**DOI:** 10.3389/fnhum.2013.00187

**Published:** 2013-05-10

**Authors:** Nicole Y. H. Yang, Dong Zhou, Raymond C. K. Chung, Cecilia W. P. Li-Tsang, Kenneth N. K. Fong

**Affiliations:** ^1^Department of Rehabilitation Medicine, West China Hospital, Sichuan UniversityChengdu, China; ^2^Institute for Disaster Management and Reconstruction, Sichuan UniversityChengdu, China; ^3^Department of Rehabilitation Sciences, The Hong Kong Polytechnic UniversityHong Kong; ^4^Department of Neurology, West China Hospital, Sichuan UniversityChengdu, China

**Keywords:** systematic review, stroke, unilateral neglect, rehabilitation, Behavioral Inattention Test

## Abstract

A systematic review of the effectiveness of rehabilitation for persons with unilateral neglect (UN) after stroke was conducted by searching the computerized databases from 1997 through 2012. Randomized controlled trials (RCTs) of neglect treatment strategies for stroke patients which used the Behavioral Inattention Test (BIT) as the primary outcome measure were eligible for inclusion. Out of 201 studies initially identified, 12 RCTs covering 277 participants were selected for analysis. All had the same weakness of low power with smaller samples and limitation in the blinding of the design. Prism Adaptation (PA) was the most commonly used intervention while continuous Theta-burst stimulation (cTBS) appeared to be a new approach. Meta-analysis showed that for immediate effects, the BIT conventional subscore had a significant and large mean effect size (ES = 0.76; 95% CI 0.28–1.23; *p* = 0.002) whereas the BIT total score showed a modestly significant mean ES (ES = 0.55; 95% CI 0.16–0.94; *p* = 0.006). No significant mean ES in sensitivity analysis was found for long-lasting effects across all BIT outcomes. PA appeared to be the most effective intervention based on the results of pooled analysis. More rigorous studies should be done on repetitive transcranial magnetic stimulation (rTMS) before it can be concluded that it is a promising treatment for UN.

## Introduction

Unilateral neglect (UN) is a heterogeneous perceptual disorder that often follows stroke, especially after right hemisphere lesion. Its most typical feature is failure to report or respond to stimuli presented from the contralateral space, including visual, somatosensory, auditory, and kinesthetic sources. Sufferers may even fail to perceive their own body parts (Mesulam, 1999). The reported incidence varies from 10 to 82% following right- and from 15 to 65% following left-hemisphere stroke (Plummer et al., 2003). Subject selection criteria, lesion site, the nature and timing of the assessment, and lack of agreement on assessment methods are all responsible for the variability in these reported rates (Stone et al., 1991; Azouvi et al., 2002). UN has a significant negative impact associated with functional recovery at home discharge (Jehkonen et al., 2006; Mutai et al., 2012).

Different treatment approaches and assessment tools have been developed to evaluate and address UN. The most recent literature shows that rehabilitation can be classified under two types of behavioral approaches: recruiting the hemiplegic limbs to reduce spatial preference for the ipsilesional space, or improving awareness of the contralesional space to promote patients’ attention (Pierce and Buxbaum, 2002; Paci et al., 2010). More than 18 methods using these general approaches have been put into practice (Luauté et al., 2006) with varying results based on a large number of outcome measures. Although the reported quality is moderate for most of the RCTs in neglect rehabilitation (Paci et al., 2010), some interventions appear to be more promising. Comments have also been made that the effects of treatment are often task-specific or transient and cannot be generalized to daily functioning (Pierce and Buxbaum, 2002; Bowen et al., 2007). Due to a lack of evidence, it is also hard to report which approach is the optimal recommendation for clinical practice (Luauté et al., 2006), and interestingly, professional therapists rarely use these scientifically proven interventions (Petzold et al., 2012).

Many RCTs have employed “pencil-and-paper” tasks, including line bisection, cancelation tasks, copying, and drawing, as treatment outcomes for UN. One of the commonest tests, and one that has been used extensively as an outcome measure for UN, is the Behavioral Inattention Test (BIT) (Bowen et al., 1999, 2007). This is a criterion-referenced test for UN or visual inattention in patients suffering from stroke or brain injuries, comprising two parts: the conventional and the behavioral subtests (Halligan et al., 1991). The conventional subtests include six traditional paper-and-pencil tasks: line crossing, letter cancelation, star cancelation, figure copying, line bisection, and representative drawing. The behavioral subtests consist of nine simulated daily living tasks: picture scanning, telephone dialing, menu reading, article reading, telling and setting the time, coin sorting, address and sentence copying, map navigation, and card sorting. Both parts can be used separately in clinical for impairment and function level assessments, and it has been recommended as a good predictor of functional performance in daily living with good construct and predictive validity (Hartmanmaeir and Katz, 1995).

The aim of this study was to develop a systematic review to assess the effectiveness of rehabilitation for UN as measured by the BIT and to evaluate the effects of the interventions reported in the RCTs using a meta-analysis.

## Methods

### Database

We searched the following electronic databases for trials published in English; PubMed/Medline (1965+ via EbscoHost), PsycINFO (1806+), physiotherapy evidence database (PEDro), Science Direct, CINAHL (Cumulative Index to Nursing and Allied Health Literature, 1982+), and Cochrane Central Register of Controlled Trials (CENTRAL). We also hand-searched the bibliographies of all studies ordered in full text. Date of publication was limited from January 1997 to June 2012 as most of the full-text electronic versions of journal papers are available since 1997.

The terms used in the search were: cerebrovascular accident OR stroke; neglect; visuo-spatial neglect; visual neglect; unilateral neglect; and hemisphere neglect. The search was limited to RCTs involving adults aged 19 or over.

### Selection criteria

We included all RCTs that sought to identify the effectiveness of any type of rehabilitation intervention in UN in adult stroke patients diagnosed by clinical examination and/or classical neuropsychological tests. Only studies which reported the BIT (Wilson et al., 1987) as the primary outcome measure were included. The BIT includes a score for the conventional subtest (BIT-C) and/or the behavioral subtest (BIT-B) as well as the total score [BIT (Total)].

We excluded observational studies and case reports as well as cross-over design studies; studies where full text was not available; studies with a sample size of less than five in each group; and those rated as 4 or less out of 10 by the PEDro in the quality assessment described below. Cross-over design studies were excluded in our review as they usually confounded the estimates of the treatment effects with carry-over and learning effects (Leslie and Mary, 2007).

### Quality assessment

After the database search, two reviewers assessed the methodological quality of the trials according to the PEDro scale. This was developed specifically for evaluating the quality of studies aiming to compare the effectiveness of rehabilitation (Verhagen et al., 1998; Sherrington et al., 2000) and has been proved to be valid in measuring the methodological quality of clinical trials. There are 11 items in the PEDro scale. The first criterion, item eligibility, is not scored as it is used as a component of external validity; the remaining items yield a total score from 10 (RCT that meets all items) to 0 (RCT that does not meet any item) (Paci et al., 2010). The PEDro scale item scores can be summed to obtain a total score that can be used as interval data for parametric statistical analysis (Bhogal et al., 2005; de Morton, 2009). The PEDro scale classifies studies as high or low quality based on a cut-off score of six (Maher et al., 2003). Articles scoring six or higher are considered of high quality and low-quality studies score less than six.

### Data extraction and analysis

Each selected study was carefully assessed against the inclusion criteria, and the necessary information and characteristics summarized in a table. We calculated Cohen’s *d* on individual treatment effect size (ES) for these studies and compared the effectiveness among different interventions. Meta-analysis on overall treatment effectiveness was done with Review Manager Version 5.0 (Copenhagen: The Nordic Cochrane Center, The Cochrane Collaboration, 2012). The standardized mean difference (SMD) was presented as the ES and its 95% confidence interval (CI) computed. Because of the heterogeneity of the interventions, we could only perform a pooling for meta-analysis for a single intervention reported in two or more trials. The test of heterogeneity was used to assess the potential heterogeneity across studies. If heterogeneity existed, a random-effect model was used. The random-effect approach assumes that the ES from each trial is a random sample from a larger population of possible ES. Otherwise, the fixed-effect model was used. A sensitivity analysis was also used to assess the impact of overall treatment effectiveness by excluding each trial once at a time.

## Results

**Figure 1:**
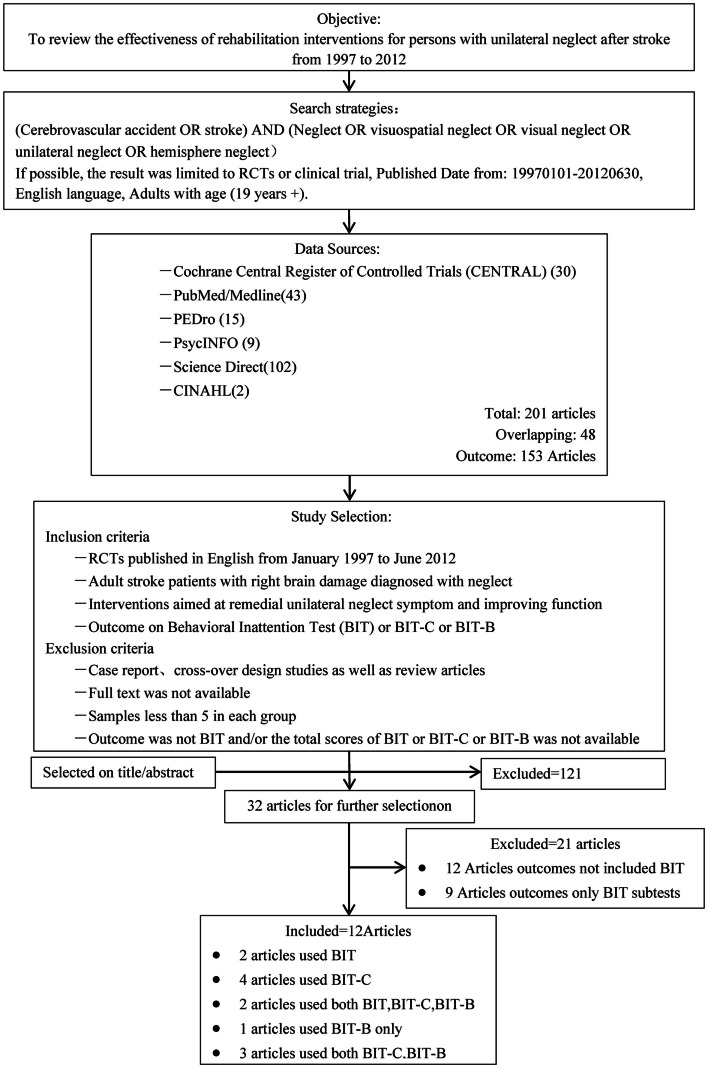
**Overview of the search and selection process**.

Figure 1 illustrates the selection process. The initial search yielded 201 citations from January 1997 through June 2012. After removing duplicates, 153 citations remained. Based on the title and abstract of the articles, 32 potentially relevant articles were selected. After careful evaluation by the reviewers, we identified 25 clinical trials (Wiart et al., 1997; Robertson et al., 2002; Harvey et al., 2003; Pizzamiglio et al., 2004; Katz et al., 2005; Fong et al., 2007; Nys et al., 2008; Schroder et al., 2008; Ertekin et al., 2009; Luukkainen-Markkula et al., 2009; Polanowska et al., 2009; Serino et al., 2009; Song et al., 2009; Tsang et al., 2009; Saevarsson et al., 2010; Turton et al., 2010; Ferreira et al., 2011; Kamada et al., 2011; Kim et al., 2011; Làdavas et al., 2011; Mizuno et al., 2011; Welfringer et al., 2011; Gorgoraptis et al., 2012; Ianes et al., 2012; Koch et al., 2012) to be included in the final assessment. Of these, 12 articles were included in our final review (Robertson et al., 2002; Harvey et al., 2003; Fong et al., 2007; Nys et al., 2008; Luukkainen-Markkula et al., 2009; Serino et al., 2009; Tsang et al., 2009; Turton et al., 2010; Ferreira et al., 2011; Làdavas et al., 2011; Mizuno et al., 2011; Koch et al., 2012) with the others excluded because the BIT was not used as the primary outcome measure.

The quality of all 12 RCTs was fair to good (Table 1). Four (33.3%) were identified as of fair quality as their scores were below six in the scale. Two studies (Mizuno et al., 2011; Koch et al., 2012) used double-blind designs whereas others were mostly single-blind.

**Table 1:** **PEDro scores of included studies**.

Studies	Eligibility	1, Random allocation	2, Concealed allocation	3, Baseline comparability	4, Blind subjects	5, Blind therapists	6, Blind assessors	7, Adequate follow-up	8, Intention-to-treat analysis	9, Between-group comparisons	10, Point estimates variability	Score	Quality
**ITEMS**													
Nys et al. (2008)	Yes	1	0	1	1	0	0	1	0	1	1	6/10	Good
Serino et al. (2009)	Yes	0	0	1	1	0	0	1	0	1	1	5/10	Fair
Turton et al. (2010)	Yes	1	1	0	0	0	1	1	0	1	1	6/10	Good
Mizuno et al. (2011)	Yes	1	1	1	1	0	1	1	0	1	1	8/10	Good
Làdavas et al. (2011)	Yes	1	0	1	1	0	1	0	0	1	1	6/10	Good
Robertson et al. (2002)	Yes	1	0	1	0	0	1	1	0	1	1	6/10	Good
Luukkainen-Markkula et al. (2009)	Yes	1	1	1	0	0	0	1	0	0	1	5/10	Fair
Fong et al. (2007)	Yes	1	0	1	0	0	1	1	0	1	1	6/10	Good
Tsang et al., 2009	Yes	1	1	1	0	0	1	0	0	1	1	6/10	Good
Harvey et al. (2003)	Yes	1	0	1	1	0	0	1	0	1	0	5/10	Fair
Koch et al. (2012)	Yes	1	1	1	1	1	1	1	0	1	1	9/10	Good
Ferreira et al. (2011)	No	1	0	1	0	0	0	1	0	1	1	5/10	Fair

### Characteristics of the studies

Descriptions of the 12 articles reviewed are listed in Table 2. A total of 277 subjects with UN were included in this analysis. All were adults with right brain damage due to stroke; most had a diagnosis of first single right hemisphere stroke. The duration from stroke onset to study covered the period from the acute (≤4 weeks) to the chronic phase (≥6 months), but most studies were conducted in the subacute and chronic phases after stroke. All studies used similar selection criteria.

**Table 2:** **Characteristics of included studies**.

Studies	Methods					Interventions			BIT results	
	Type	Study design	Control	Groups subjects (*n*)	Duration from onset to treatment	Treatment	Regime	Duration	Immediate	Long-term
Nys et al. (2008)	PA	single-blind RCT	Placebo (neutral goggles)	*n* = 16 PA gp = 10 CT gp = 6	≤4 weeks	Wore pair of goggles fitted with wide-field point-to-point prismatic lenses shifted their visual field 10°/0° rightward and do some fast pointing movements	30 min/session 4-days-in-row sessions	4 days		BIT-C (−); BIT-B (−); follow-up = 1 month
Serino et al. (2009)	PA	single-blind pseudo-RCT	Placebo (neutral goggles)	*n* = 20 PA gp = 10 CT gp = 10	≥1 month	Wore prismatic lenses, which shifted their visual field 10°/0°rightward and pointing movements	30 min/Session 10 daily sessions within 2 weeks	2 weeks	BIT (+)	BIT (+); follow-up = 1 month
Turton et al. (2010)	PA	single-blind RCT	Placebo (flat plain glass)	*n* = 36 PA gp = 17 CT gp = 19 (1 drop-out) (1 drop-out)	≥20 days	Wore 10 diopter, 6 degree prisms using index finger to touch a bold vertical line on screen	Once a day, each working day	2 weeks	BIT (−)	BIT (−); follow-up = 8 weeks
Mizuno et al. (2011)	PA	double-masked RCT	Placebo (neutral glasses)	*n* = 38 PA gp = 18 CT gp = 20	≤3 months	Wore prism glasses shifted visual field 12° to right and repeat pointing tasks	20 min/Session bid, 5 days/week	2 weeks	BIT-C (−); BIT-B (−)	BIT-C (−); BIT-B (−); follow-up until discharge
Làdavas et al. (2011)	PA	single-blind pseudo-RCT	Placebo (neutral glasses)	*n* = 30 TPA gp = 10 CPA gp = 10 CT gp = 10	≥2 months	Wore wide-field prismatic lenses inducing a 10° shift visual field to right and repeat pointing tasks	30 min/Session one per day, 10 sessions	2 weeks	TPA:BIT-B (+); BIT-C (+); CPA:BIT-C (−); BIT-B (−)	No follow-up
Robertson et al. (2002)	LA	single-blind RCT	Dummy device	*n* = 40 LA + PT = 19 (2 drop-out) PT = 21 (2 drop-out)	LA: 152.8 ± 142.4 PT: 152.1 ± 117.9	Using a semi-automatic device for limb activation combined with perceptual training	45 min/Session once a week 12 sessions	12 weeks	BIT-B (−)	BIT-B (−); follow-up = 18–24 months
Luukkainen-Markkula et al. (2009)	LA	single-blind RCT	Conventional visual scanning training	*n* = 12 LA gp = 6 CT gp = 6	≤6 months	Arm activation training (determined by the individual hand and arm motor status assessed by WMFT)	Total 48 h of therapy	3 weeks	BIT-C (+)	BIT-C (+) follow-up = 6 months
Fong et al. (2007)	TRTR + EP	single-blind RCT	Conventional OT	*n* = 54 TR gp = 19 TR + EP gp = 20 CT gp = 15	≤8 weeks	Trunk rotation was performed in three different positions: supine lying on a plinth, unsupported sitting on a plinth, and standing in a standing frame	1 h/Session 5 times/week	30 days	BIT-B (−); BIT-C (−); BIT (−)	BIT-B (−); BIT-C (−); BIT (−); follow-up = 60 days
Tsang et al. (2009)	EP	single-blind RCT	Conventional OT	*n* = 34 EP gp = 17 CT: 22.18 ± 15.87	EP: 21.5 ± 21.67	Underwent occupational therapy with special glasses blocking the right half visual field	30 min ADL +30 min NDT for UL/day	4 weeks	BIT-C (+)	No follow-up
				CT gp = 17	
Harvey et al. (2003)	VF	RCT	Same activities but without feedback	*n* = 14 VF gp = 7 CT gp = 7	5–25 months	Experimenter-administered practice of rod lifting with judge center grids for proprioceptive and visual feedback	1 h/day with 3 days, then 10 days of home-based intervention	3 days/2 weeks	BIT-C (+); BIT-B (−)	BIT-C (+); BIT-B (−); follow-up = 1 month
					
Koch et al. (2012)	TBS	double-blind RCT	Sham coil angled 90°	*n* = 18 TBS gp = 9 CT gp = 9	≥1 months (43 ± 16 days)	3-pulse bursts at 50 Hz repeated every 200 ms for 40 s, 80% AMT over the left PPC	2 Sessions/day, 15 min interval; 5 days/week	2 weeks	BIT-B (+); BIT-C (+); BIT (+)	BIT-B (+); BIT-C (+); BIT (+); follow-up = 1 month
Ferreira et al. (2011)	MP VST	single-blind RCT	Conventional PT without any treatment for neglect	*n* = 15 MP gp = 5 VST gp = 5 CT gp = 5	≥3 months	VS: the protocol included 4 tasks: 2 directed to the extrapersonal space and 2 addressing peripersonal neglect; MP: included 4 tasks: 2 tasks of motor imagery and 2 of visual imagery	1 h/Session twice per week	5 weeks	VST: BIT-C (+); MP: BIT-C (−)	VST: BIT-C (+); MP:BIT-C (−) follow-up = 2 months

*PA, prism adaptation; LA, limb activation; TR, trunk rotation; EP, eye patching; VF, visuomotor feedback; TBS, theta-burst stimulation; MP, mental practice; VST, visual scanning training; BIT (Total), total score on Behavioral Inattention Test; BIT-C, BIT conventional subtest; BIT-B, BIT behavioral subtest; OT, occupational therapy; PT, physiotherapy*.

Among the 12 studies, 5 (Nys et al., 2008; Serino et al., 2009; Làdavas et al., 2011; Mizuno et al., 2011) studied the effectiveness of prism adaptation (PA). There were differences in the PA procedure used; one study (Nys et al., 2008) used repetitive PA for a short period while another used different feedback strategies in PA (terminal and concurrent prism adaptation). During terminal PA, only the final part of the pointing movement is visible and PA relies most strongly on a strategic recalibration of visuomotor eye–hand (Làdavas et al., 2011). In contrast, in concurrent PA the second half of the pointing movement is visible, and thus adaptation mainly consists of a realignment of proprioceptive coordinates (Làdavas et al., 2011). All five studies used the same control methods with neutral goggles. Two articles (Robertson et al., 2002; Luukkainen-Markkula et al., 2009) applied limb activation. Other studies used different interventions; visuomotor feedback, virtual reality, repetitive transcranial magnetic stimulation (rTMS), and continuous Theta-burst stimulation (cTBS). Compared to a previous review (Luauté et al., 2006), no new intervention was reported in our review during the time period stated except for cTBS. All studies investigated a single treatment, except for one RCT (Fong et al., 2007) which investigated the effectiveness of a combination of two different methods, namely trunk rotation and eye patching.

The duration of treatment ranged from 4 days (Nys et al., 2008) to 5 weeks (Ferreira et al., 2011), but for half of the studies was 30 min per session for 5 sessions per week over 2 weeks, giving a total of 10 sessions. All the trials were conducted in hospitals except for one (Harvey et al., 2003) which involved self-administered home-based practice for 2 weeks.

Apart from the BIT, the outcome for neglect severity included the Catherine Bergego Scale (CBS), the Bell Cancelation Test, reading, computerized visual search tasks, and paper-and-pencil neglect tests. In all studies, functional outcomes were included, namely the Functional Independence Measure, the Barthel Index, upper limb motor functions (the Wolf Motor Function Test and the Modified Motor Assessment Scale), and the Stroke Impairment Assessment Set.

Three studies (Serino et al., 2009; Turton et al., 2010; Ferreira et al., 2011) used the BIT (Total) only; three (Nys et al., 2008; Làdavas et al., 2011; Mizuno et al., 2011) used both the BIT-C and the BIT-B separately as outcomes; and two (Fong et al., 2007; Koch et al., 2012) used the BIT (Total) and both the BIT-C and BIT-B as outcomes. Only one study (Robertson et al., 2002) used only the BIT-B as the outcome.

#### Effects of rehabilitation interventions

**Figure 2:**
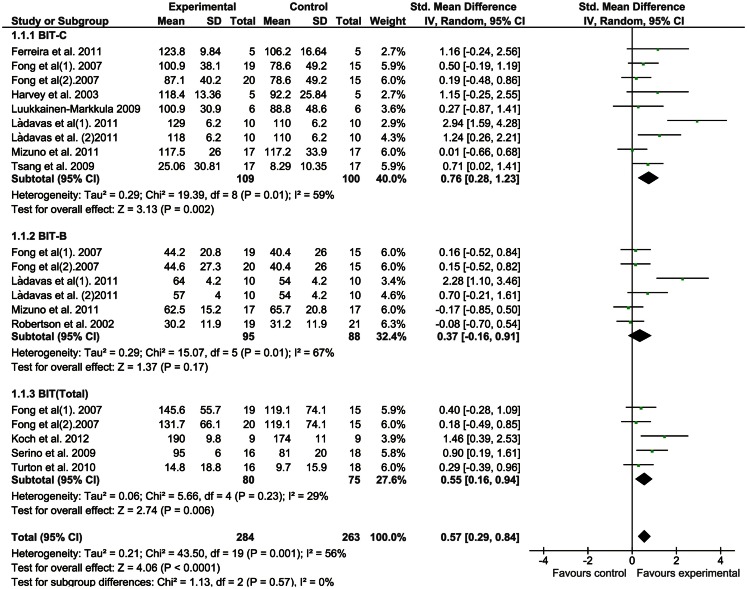
**Rehabilitation interventions versus any control, outcome: immediate effects**.

**Figure 3:**
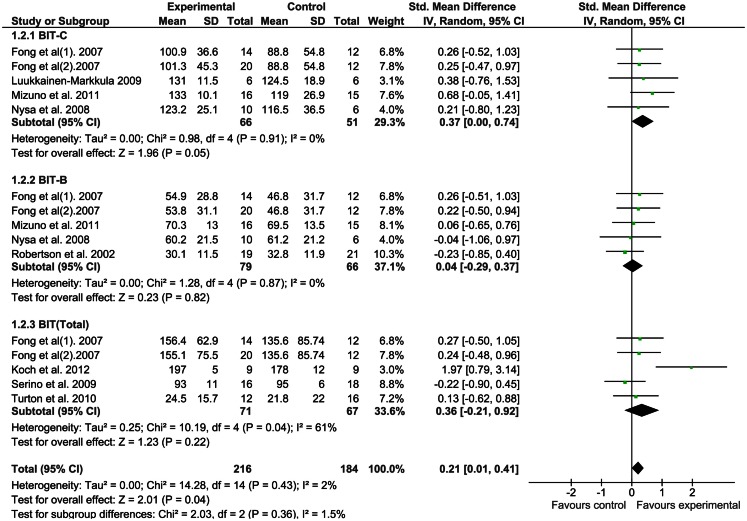
**Rehabilitation interventions versus any control, outcome: long-lasting effects**.

We applied a meta-analysis on all outcomes to calculate SMD and 95% CI using random-effects models. A comparison of the results of both the immediate and long-lasting effects is presented in forest plots (Figures 2 and 3).

#### Immediate effects of interventions

Figure 2 shows the forest plot of the immediate effects of the interventions covered in the 12 studies. The meta-analysis shows that there was significant heterogeneity across the studies, so the random-effect model was chosen. The BIT-C had a significant mean ES of 0.76 (95% CI, 0.28–1.23; *p* = 0.002). The BIT-B showed an insignificant mean ES of 0.37 (95% CI, −0.19 to 0.91; *p* = 0.17), and the BIT (Total) a statistically significant mean ES of 0.55 (95% CI, 0.16–0.94; *p* = 0.006). The sensitivity of each trial on the mean ES was also assessed by excluding each trial one at a time. The overall results were the same even when any single trial was eliminated.

#### Long-lasting effects of rehabilitation interventions

Figure 3 shows the forest plot of the long-lasting effects of the interventions studied. The meta-analysis shows that none of the ES were significant for the BIT outcomes except the BIT-C (*p* = 0.05). The sensitivity of each trial on the mean ES was also evaluated by excluding one trial at a time, but the results were not significant (*p* > 0.05).

To find out the optimal intervention for UN, Cohen’s *d* was calculated on the individual ES of each approach as the difference between the pre- and posttest means for the single treatment group divided by the SD of the pretest scores. There was more than one paper covering PA, so we pooled the ES of PA in three studies for the BIT-C, two for the BIT-B, and two for the BIT (Total) before conducting a relative comparison of the ES of all studies. The results showed that for immediate effects, after pooling, PA had the highest ES as measured by the BIT-C and the BIT-B, while cTBS had the highest ES measured by the BIT (Total). All interventions showed low ES for long-lasting effects (Tables 3 and 4).

**Table 3:** **Immediate effect size of each rehabilitation intervention**.

Outcomes	Study	Intervention	Effect size
BIT-C	Làdavas et al. (2011) (1)	PA	1.31 (−0.26, 2.88) (pooled)
	Làdavas et al. (2011) (2)		
	Mizuno et al. (2011)	
	Ferreira et al. (2011)	VST	1.16 (−0.24, 2.56)
	Harvey et al. (2003)	VF	1.15 (−0.25, 2.55)
	Tsang et al. (2009)	EP	0.71 (0.02, 1.41)
	Fong et al. (2007) (1)	TR	0.50 (−0.19, 1.19)
	Luukkainen-Markkula et al. (2009)	LA	0.27 (−0.87, 1.41)
	Fong et al. (2007) (2)	TR + EP	0.19 (−0.48, 0.86)
BIT-B	Làdavas et al. (2011) (1)	PA	0.86 (−0.45, 2.18) (pooled)
	Mizuno et al. (2011)		
	Fong et al. (2007) (1)	TR	0.16 (−0.52, 0.84)
	Fong et al. (2007) (2)	TR + EP	0.15 (−0.52, 0.82)
	Robertson et al. (2002)	LA	−0.08 (−0.70, 0.54)
BIT (Total)	Koch et al. (2012)	TBS	1.46 (0.39, 2.53)
	Serino et al. (2009)	PA	0.55 (0.16, 0.94) (pooled)
	Turton et al. (2010)		
	Fong et al. (2007) (1)	TR	0.40 (−0.28, 1.09)
	Fong et al. (2007) (2)	TR + EP	0.18 (−0.49, 0.85)

**Table 4:** **Long-lasting effect size of each rehabilitation intervention**.

Items	Study	Intervention	Effect size
BIT-C	Mizuno et al. (2011)	PA	0.52 (−0.07, 1.11) (pooled)
	Nys et al. (2008)		
	Luukkainen-Markkula et al. (2009)	LA	0.38 (−0.76, 1.53)
	Fong et al. (2007) (1)	TR	0.26 (−0.52, 1.03)
	Fong et al. (2007) (2)	TR + EP	0.25 (−0.47, 0.97)
BIT-B	Fong et al. (2007) (1)	TR	0.26 (−0.51, 1.03)
	Fong et al. (2007) (2)	TR + EP	0.22 (−0.50, 0.94)
	Mizuno et al. (2011)	PA	0.03 (−0.55, 0.60) (pooled)
	Nys et al. (2008)		
	Robertson et al. (2002)	LA	−0.23 (−0.85, 0.40)
BIT (Total)	Fong et al. (2007) (1)	TR	0.27 (−0.50, 1.05)
	Fong et al. (2007) (2)	TR + EP	0.24 (−0.48, 0.96)
	Koch et al. (2012)	TBS	1.97 (0.79, 3.14)
	Serino et al. (2009)	PA	−0.06 (−0.57, 0.44) (pooled)
	Turton et al. (2010)		

#### Pooled effects of PA on UN

The pooled ES of the single intervention PA on each BIT outcome were also analyzed (Table 5). No statistically significant results were found for either immediate or long-lasting effects as reflected in the BIT outcomes with significant heterogeneity.

**Table 5:** **PA intervention on neglect**.

Outcome or subgroup	Studies	Participants	Statistical method	Effect estimate
Immediate effects	5	216	Std. mean difference (IV, random, 95% CI)	0.89 (0.27, 1.51)
BIT-C	3	74	Std. mean difference (IV, random, 95% CI)	1.31 (−0.26, 2.88)
BIT-B	3	74	Std. mean difference (IV, random, 95% CI)	0.86 (−0.45, 2.18)
BIT (Total)	2	68	Std. mean difference (IV, random, 95% CI)	0.59 (−0.02, 1.19)
Long-lasting effects	4	125	Std. mean difference (IV, random, 95% CI)	0.15 (−0.20, 0.51)
BIT-C	2	47	Std. mean difference (IV, random, 95% CI)	0.52 (−0.07, 1.11)
BIT-B	1	16	Std. mean difference (IV, random, 95% CI)	−0.04 (−1.06, 0.97)
BIT (Total)	2	62	Std. mean difference (IV, random, 95% CI)	−0.06 (−0.57, 0.44)

## Discussion

Our systematic review indicates that there is modest evidence for the use of PA to reduce UN in stroke, with immediate and long-lasting effects, and eye patching as shown by BIT-C scores for immediate effects. Other studies obtained positive effects from the use of visual scanning training (Ferreira et al., 2011), visuomotor feedback (Harvey et al., 2003), and TBS (Koch et al., 2012). Since Koch et al. (2012) only report the BIT (Total) and not the BIT-C and BIT-B subscale scores, it is impossible to draw any conclusion that rTMS is better than PA in improving the performance of tasks in the BIT-C and the BIT-B for neglect patients as no comparison could be done.

According to this review, PA is inclined to exhibit the highest ES for immediate effects, but this was not statistically significant as the 95% CI crossed over the zero point. The possible neural mechanism underlying the therapeutic effect of PA is that it reduces spatial neglect by enhancing the recruitment of intact brain areas responsible for visuo-spatial output through short-term sensori-motor plasticity pathways (Rossetti et al., 1998; Luauté et al., 2006). Although this technique has produced some improvement in a wide range of neglect symptoms, especially visual (Shiraishi et al., 2010; Mizuno et al., 2011; Rusconi and Carelli, 2012), some contradictory results have also been reported (Ferber et al., 2003; Rousseaux et al., 2006). The inconsistent results are probably due to the lack of comparability of treatment apparatus, treatment duration, the tasks used to assess PA effects, and post-stroke duration. Similar to PA, hemiplegic half-field eye patching is another compensational intervention for neglect which works by blocking the ipsilesional visual field. The initial study by Tsang et al. (2009) demonstrates a significant result with an ES of 0.71 immediately after intervention. More good-quality RCTs are needed to assess its long-lasting effects on UN.

Transcranial magnetic stimulation is a safe and non-invasive procedure to detect or modulate brain activity by passing a strong brief electrical current through an insulated wired coil placed on the skull which generates a transient magnetic field in the brain (Hummel and Cohen, 2006). TBS is a kind of rTMS using a lower stimulation intensity and a shorter time of stimulation to induce long-lasting effects in the cortex (Cárdenas-Morales et al., 2010) which demonstrates a relatively high ES as measured by the BIT total scores discussed in this review. TMS has become a popular method to stimulate the human brain, with rTMS attracting particular interest for its therapeutic potential to modify cortical excitability (Funke and Benali, 2011), which sheds light on the use of the inter-hemispheric rivalry model in explaining the recovery after neglect disorder in stroke patients. According to the literature, rTMS induces and repairs the inter-hemispheric imbalance (a neglect-like behavior) in the left or right posterior parietal cortex in healthy humans (Kinsbourne, 1977, 1994; Oliveri et al., 2001; Rounis et al., 2007). Based on this model, some studies have explored whether the use of inhibitory rTMS over the contralesional hemisphere to reduce the pathological hyperactivity of either hemisphere may be useful in promoting recovery from neglect after stroke with promising results (Oliveri et al., 2001; Brighina et al., 2003; Shindo et al., 2006; Koch et al., 2008; Nyffeler et al., 2009; Song et al., 2009). Compared to traditional standard cognitive intervention, rTMS can accelerate clinical recovery (Oliveri et al., 2001; Shindo et al., 2006; Song et al., 2009; Paik and Paik, 2010). It seems that patients more severely affected at baseline also benefited more from this intervention. However, the small sample size of the TBS study makes it impossible to draw any conclusion based on robust evidence. There may be a publication bias whereby large studies will report small ES whereas small studies will report large ES.

This review cannot determine the best time to commence neglect rehabilitation interventions, because most participants in the studies included here were recruited in either the subacute or chronic phases. Only two studies implemented rehabilitation within 1 month of stroke (Fong et al., 2007; Nys et al., 2008). As most of the spontaneous recovery after stroke happens in the first month (Kerkhoff and Schenk, 2012), further research is necessary to determine the effects of early but specific intervention for UN compared to conventional rehabilitation in order to avoid the confounding effect of spontaneous recovery. Neglect is the best single predictor of long-term functional impairment and poor rehabilitation outcome in the early stage (Jehkonen et al., 2001; Nys et al., 2005). One study (He et al., 2007) based on neuroimaging shows that 2 weeks after stroke, the normally functional connectivity between the left and right dorsal parietal cortex was disrupted, with the degree of breakdown correlated with the severity of left spatial neglect. It is therefore reasonable that patients should start a neglect intervention as soon as possible in the acute stage, in order to avoid non-use of the hemiplegic limbs, by increasing multisensory inputs or stimulation to the ipsilateral brain regions, and thus slowing down the secondary changes in the brain related to neglect. For further research, we also recommend adequate follow-up to maximize the benefits and monitor the persistence of the effect of neglect rehabilitation interventions.

### Limitations of the review

The review has some limitations. It is constrained by the quality of the studies included, none of which scored the intention-to-treat analysis. The blindness design was the biggest weakness of most of these RCTs. The heterogeneity of the studies means that this meta-analysis is less powerful and cannot identify conclusively the optimal treatment approach.

## Conclusion

The results of this review confirm that PA appears to be the most common and effective rehabilitation intervention for UN, and that rTMS might be a promising approach for future treatment. As shown by the insignificant long-lasting effects, rehabilitation interventions often had a transient impact and could not be generalized across time to an improvement in daily functioning. All studies faced the same weakness of low power with smaller samples and a limitation in the blindness design. More rigorous studies of various interventions should be done before coming to a firm conclusion.

## Conflict of Interest Statement

The authors declare that the research was conducted in the absence of any commercial or financial relationships that could be construed as a potential conflict of interest.
